# Seasonal and spatial patterns of eukaryotic phytoplankton communities in an urban river based on marker gene

**DOI:** 10.1038/s41598-021-02183-5

**Published:** 2021-11-30

**Authors:** Jing Yang, Junping Lv, Qi Liu, Fangru Nan, Bo Li, Shulian Xie, Jia Feng

**Affiliations:** 1 School of Life Science, Shanxi University, Taiyuan, 030006 China; 2 School of Geographical Science, Taiyuan Normal University, Jinzhong, 030619 China

**Keywords:** Community ecology, Freshwater ecology, Microbial ecology

## Abstract

The seasonal and spatial eukaryotic phytoplankton composition in the Fenhe River was investigated based on the 18S rDNA V4 region. The relationship between phytoplankton functional groups and environmental factors was explored to effectively capture the responses of these taxa to environmental gradients and their effects on ecosystem function. Our results indicated that the Chlorophyta and Bacillariophyta had higher relative abundance than other taxa, and their diversity and richness indices in spring were higher than those in other seasons. The linear discriminant analysis effect size (LEfSe) analyses detected that the potential seasonal biomarkers included *Desmodesmus*, *Cyclotella*, *Pseudoschroederia*, *Discostella*, *Scenedesmus*, *Monoraphidium*, and *Nannochloropsis*; the spatial biomarkers included *Amphora*, *Neochloris*, *Hindakia*, *Pseudomuriella*, *Coccomyxa*, *Chloroidium*, *Scherffelia*, *Chromochloris*, and *Scotinosphaera*. The systemic evolution and distribution characteristics of the first 50 representative sequences showed that the dominant genus included *Desmodesmus* in spring, *Pseudopediastrum* in summer, *Mychonastes* in autumn, and *Monoraphidium* in winter. Main seasonal variation of phytoplankton functional groups was as follows: spring (J + F + C + X1) → summer (J + F + X1 + X2) → autumn (J + F + X1 + C) → winter (X1 + J + B + X2). Pearson correlation, redundancy analysis, and variance partitioning analysis showed temperature and phosphate were the determining factors causing the changes of phytoplankton functional groups and community composition in the Fenhe River.

## Introduction

The process of urbanization can affect water bodies both qualitatively and quantitatively and can lead to a series of processes that are collectively referred to as “urban river syndrome”^1^. The increase of population density and the changes of land cover can threaten basic ecosystem services^2^, causing unprecedented loss of habitat and biodiversity, affecting public health, and posing challenges to water management^3^. In order to deal with these impacts, new monitoring methods and public participation are needed to restore the ecology and integrate environments with society and economy^4^. The Fenhe River is the second largest tributary of the Yellow River in China; it is located in Shanxi Province, which has a semi-arid climate. Over the past few decades, streamflow has been continuously decreasing because of the impact of human activities and climate change, and the river ecosystems are experiencing a rapid loss of biodiversity and ecosystem integrity, thus have become one of the subjects that have been extensively studied^5,6^. However, our understanding of the geographical distribution pattern of microbial communities and the ecological factors affecting the diversity in urban river is still very limited.

Phytoplankton is an important organismal group that can indicate the changes in aquatic environment because of its sensitivity to the changes in physical, biological, and chemical structure of aquatic ecosystems^7,8^, they mainly involve in the degradation of natural and man-made residues and nutrient cycling, and play an important role in many biogeochemical processes^9^. Previous studies have shown that phytoplankton does not usually show strong diffusion restrictions^10,11^, indicating that its distribution is more likely influenced by local environmental variables. These variables can include nutrient concentrations, light conditions^12^, water temperatures^13^, pH, and predation by grazers and zooplankton^14^, all of which are the main factors affecting the growth and reproduction of algae in water bodies. Environmentally driven changes in phytoplankton diversity can also have a significant impact on the stability and function of food webs. Therefore, the understanding of factors determining the phytoplankton communities has become one of the biggest challenges in ecology.

The development of molecular biotechnological technology based on amplicon pyrosequencing has provided a new way to study phytoplankton communities in environments. The small subunits of the ribosome RNA gene (18S rRNA gene) are present in a wide range of living cells and are highly conserved. These subunits contain conserved regions and variable regions^15^, making the 18S rRNA gene one of the most commonly used genes. The structure of the 18S rRNA gene has been described as containing nine highly variable regions, V1 to V9, of which the V4 and V9 regions are commonly used in environmental studies^16^. A study has suggested that species richness and community composition estimated based on the V4 region are similar to those estimated using full-length ribosomal RNA genes^17^; therefore, the V4 region is suitable for environmental studies.

Given that phytoplankton is a highly diverse group of photosynthetic organisms^18^, functional groups are commonly used to classify phytoplankton species to avoid losing important characteristics and responses^19–21^. The definition of functional groups can bring together different species with common functional characteristics that respond to environmental changes in a similar way and have the same impact on ecosystem function^22^. Besides, the functional groups can more validly assess the response of phytoplankton communities to the environmental changes^23^. Therefore, in biodiversity research, the combination of species diversity and its functions is particularly useful for the understanding of the patterns of diversity and their underlying mechanisms.

In this study, we investigated the phytoplankton diversity and functional groups using 18S rDNA V4 region, with related physical and chemical variables (including water temperature, carbon, nitrogen, and phosphorus) in the Fenhe River. We combined the functional group theory with the phytoplankton diversity succession and attempted to reveal the biogeographical distribution and seasonal migration of phytoplankton and identify possible driving mechanisms based on environmental factors and phytoplankton functional groups in the Fenhe River.

## Results

### Effects of seasonality on physiochemical properties of water

**Figure 1:**
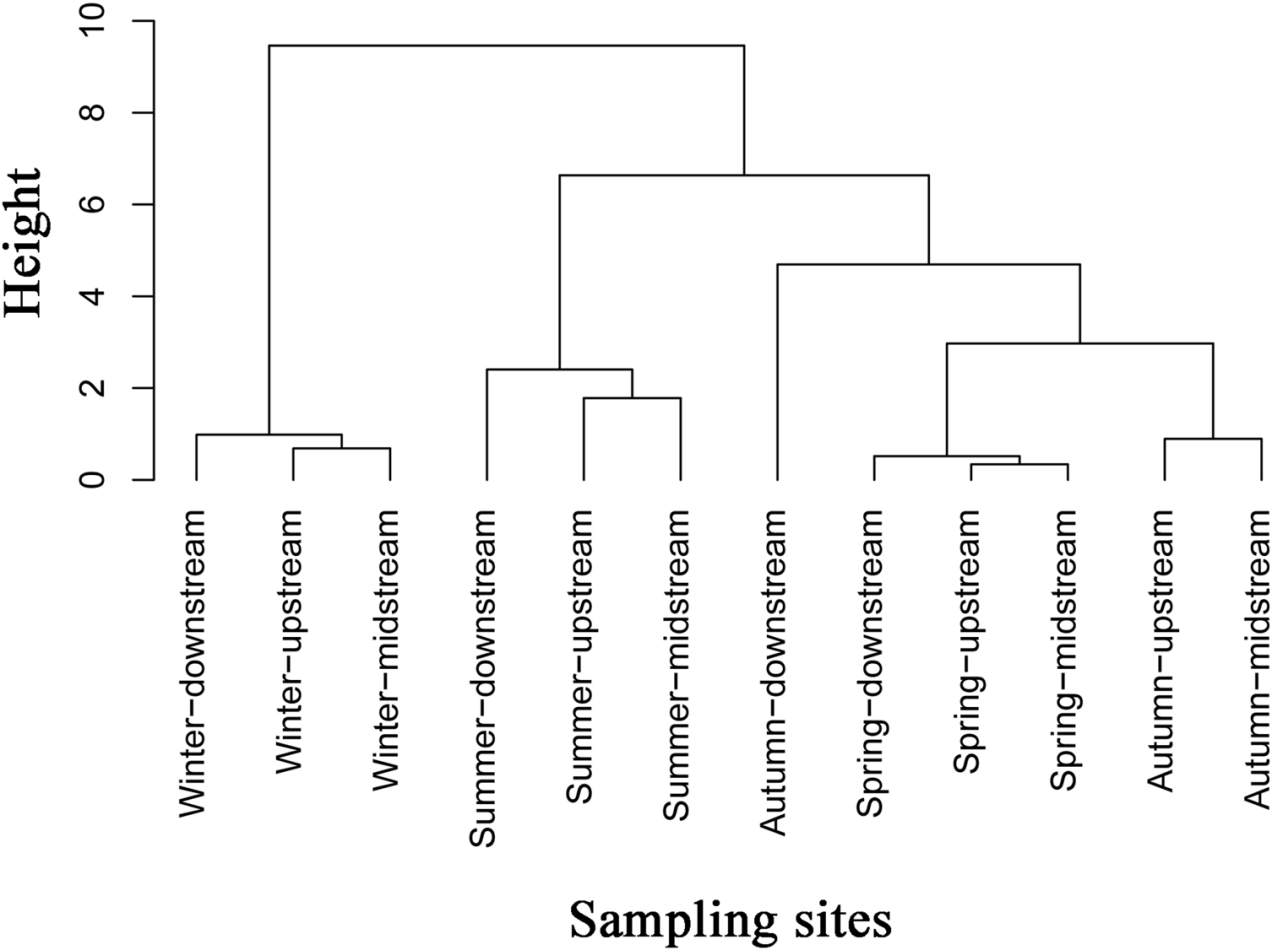
Dendrogram of environmental variables data with hierarchical cluster analysis based on the Euclidean distance and Ward linkage. Each season is divided into upstream, midstream, and downstream in spatially.

The characteristics of the physical and chemical factors of the river are shown in Table 1. Variance analysis showed that the environmental conditions during the four seasons were significantly different (*P* < 0.01), indicating the seasonal changes can affect the water quality in the Fenhe River. Although the spatial changes were not significantly different, the samples from downstream sites were separated from those from the upstream and midstream sites during the four seasons (Fig. 1). In addition, the levels of nitrite, phosphate, and DOC in the upstream sites were lower than those in the downstream sites, suggesting that the water quality in the former was better than that in the latter.

**Table 1:** One-way ANOVA analysis of physiochemical parameters in seasonally. *x̅* ± s (s.d.) is expressed as mean ± standard deviation, and *P*-value less than 0.05 is statistically significant.

Environmental variable	Season	*x̅* ± s (s.d.)	*F*-value	Anova *P*-value
Water temperature (℃)	Spring	20.283 ± 1.053	1573.605	0.001
	Summer	22.983 ± 0.256		
	Autumn	21.150 ± 0.259		
	Winter	3.167 ± 0.234		
Nitrate (mg/L)	Spring	1.572 ± 0.264	9.172	0.001
	Summer	1.268 ± 0.551		
	Autumn	0.735 ± 0.620		
	Winter	0.341 ± 0.169		
Nitrite (mg/L)	Spring	0.035 ± 0.006	37.247	0.000
	Summer	0.145 ± 0.034		
	Autumn	0.043 ± 0.024		
	Winter	0.035 ± 0.096		
Phosphate (mg/L)	Spring	0.095 ± 0.009	18.223	0.000
	Summer	0.160 ± 0.033		
	Autumn	0.062 ± 0.027		
	Winter	0.059 ± 0.031		
DOC (mg/L)	Spring	6.770 ± 0.220	176.402	0.000
	Summer	6.198 ± 0.457		
	Autumn	6.282 ± 0.293		
	Winter	1.181 ± 0.773

### Phytoplankton diversity and richness analysis

**Figure 2:**
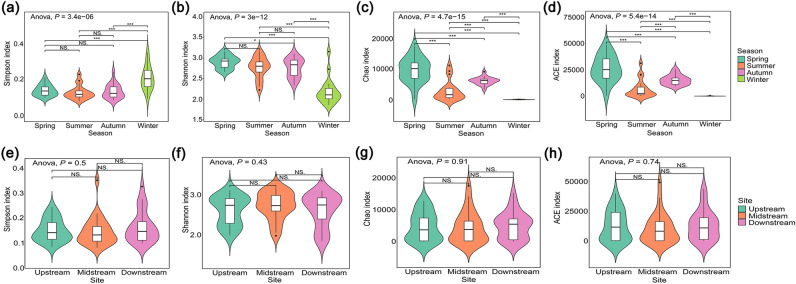
Boxplot of diversity index and richness based on one-way ANOVA. Upper row is the season variation, lower row is the spatial variation. Significant *P*-values in post-hoc test are designated with star notation: ***P < 0.001, **P < 0.01, *P < 0.05, and *NS* not significant.

The total raw reads of all samples ranged from 782,712 (autumn) to 1,547,327 (summer). After removing chimera, the number of reads in each sample ranged from 41,526 to 85,363, with a mean value of 61,629. The temporal and spatial richness estimates and diversity indices are shown in Fig. 2. The Simpson diversity index ranged between 0.08 and 0.35 with an average value of 0.15 ± 0.06, and the Shannon diversity index varied from 1.86 to 3.17 with a mean value of 2.65 ± 0.37. The Shannon and Simpson diversity indices of winter were significantly different (*P* < 0.001) from those of other seasons, whereas the Chao and ACE indices of all the four seasons were significantly different (*P* < 0.001). Overall, the diversity value in summer and autumn was equivalent, while that in winter was lowest due to its lower temperature, which is not suitable for the growth of algae. Although there were no obvious differences between the sampling sites, the upstream sites had high Shannon index but low Simpson index in spring, indicating that phytoplankton communities in these sites had high diversity.

### Differences in phytoplankton communities analyzed based on LEfSe

**Figure 3:**
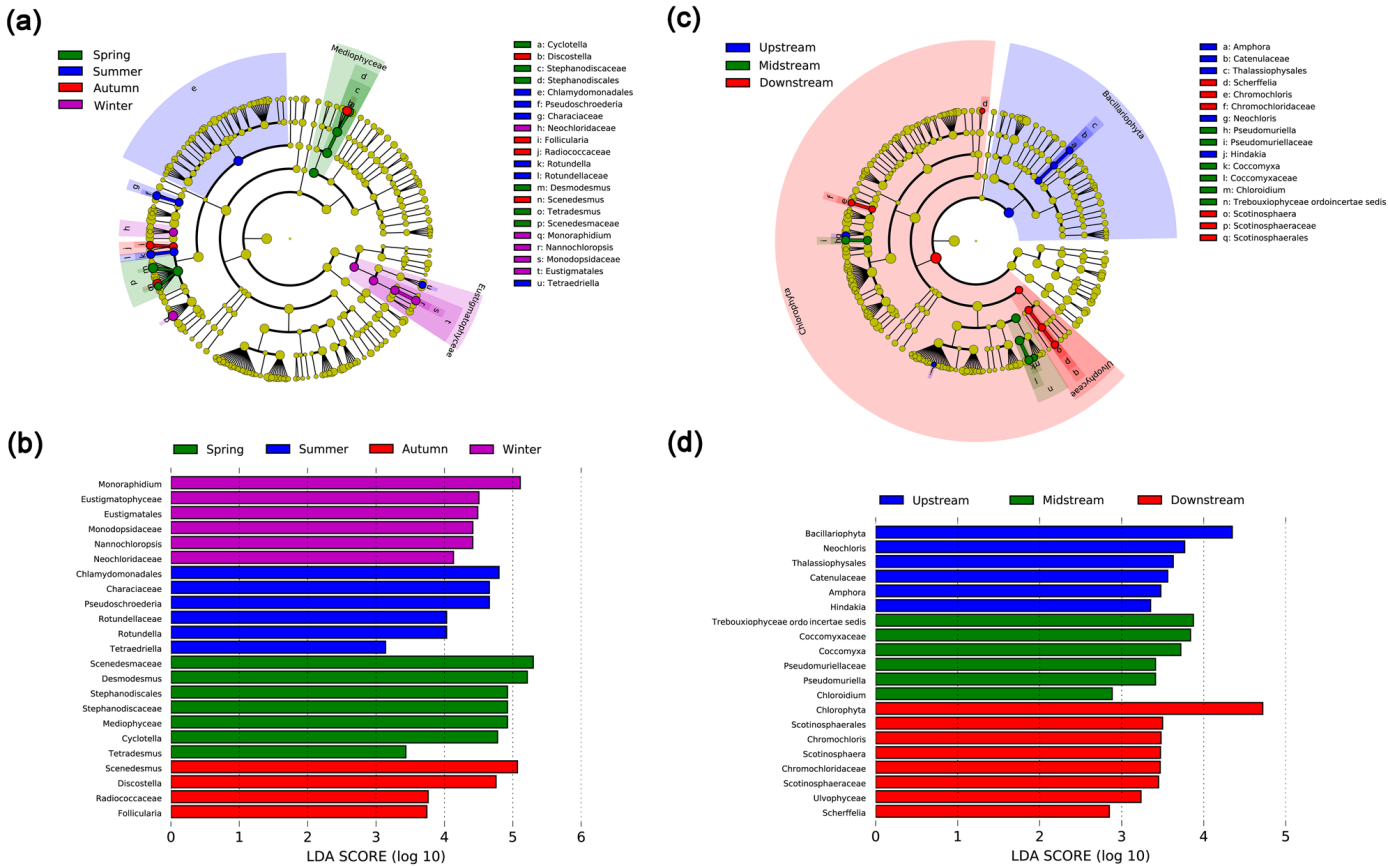
Linear discriminant analysis effect size (LEfSe) of the eukaryotic phytoplankton communities with an LDA score higher than 2.0 and *P* values less than 0.05. Each successive circle represents a phylogenetic level. Colour regions indicate taxa enriched in the different plant compartments. Bar graph shows LDA scores for phytoplankton taxa. Only taxa meeting an LDA significant threshold > 2.0 are shown. (**a**,**b**) reveal the season, (**c**,**d**) reveal the sampling sites.

The seasonal and spatial differentially abundant taxa (i.e., the biomarkers) from the phylum to the genus level identified by LEfSe analysis are shown in Fig. 3. The linear discriminant analysis (LDA) score describes the degree to which the relative abundance of various microbial groups in given microbial communities consistently changes between seasons^24^. Our results emphasized that family and genus were main the potential biomarkers in different seasons were identified and found four to seven phytoplankton taxa unique to each season. The phytoplankton groups that were enriched in spring included Mediophyceae, Stephanodiscales, Scenedesmaceae, *Desmodesmus*, and *Cyclotella*, and their LDA scores were higher than 4.5. The order Chlamydomonadales, the family Characiaceae, and the genus *Pseudoschroederia* were the biomarkers that preferred summer; their LDA scores were > 4.5. Fewer phytoplankton groups were enriched in autumn, *Discostella* and *Scenedesmus*; their LDA scores were higher than 4.5. The taxa enriched in winter samples mainly included Eustigmatales at the order level, Neochloridaceae and Monodopsidaceae at the family level, *Monoraphidium* and *Nannochloropsis* at the genus level. In spatial analysis, the biomarkers enriched at the upstream sites included the Thalassiophysales at the order level, Catenulaceae at the family level, *Amphora*, *Neochloris*, and *Hindakia* at the genus level. Trebouxiophyceae ordo incertae sedis (order), Coccomyxaceae and Pseudomuriellaceae (families), *Pseudomuriella*, *Coccomyxa*, and *Chloroidium* (genus) were enriched in the midstream sites. The downstream sites were mainly enriched with the members of the phyla Chlorophyta, the order Scotinosphaerales, the families Chromochloridaceae and Scotinosphaeraceae, and the genera *Scherffelia*, *Chromochloris*, and *Scotinosphaera*.

### Phylogenetic analysis and distribution characteristics of phytoplankton communities

**Figure 4:**
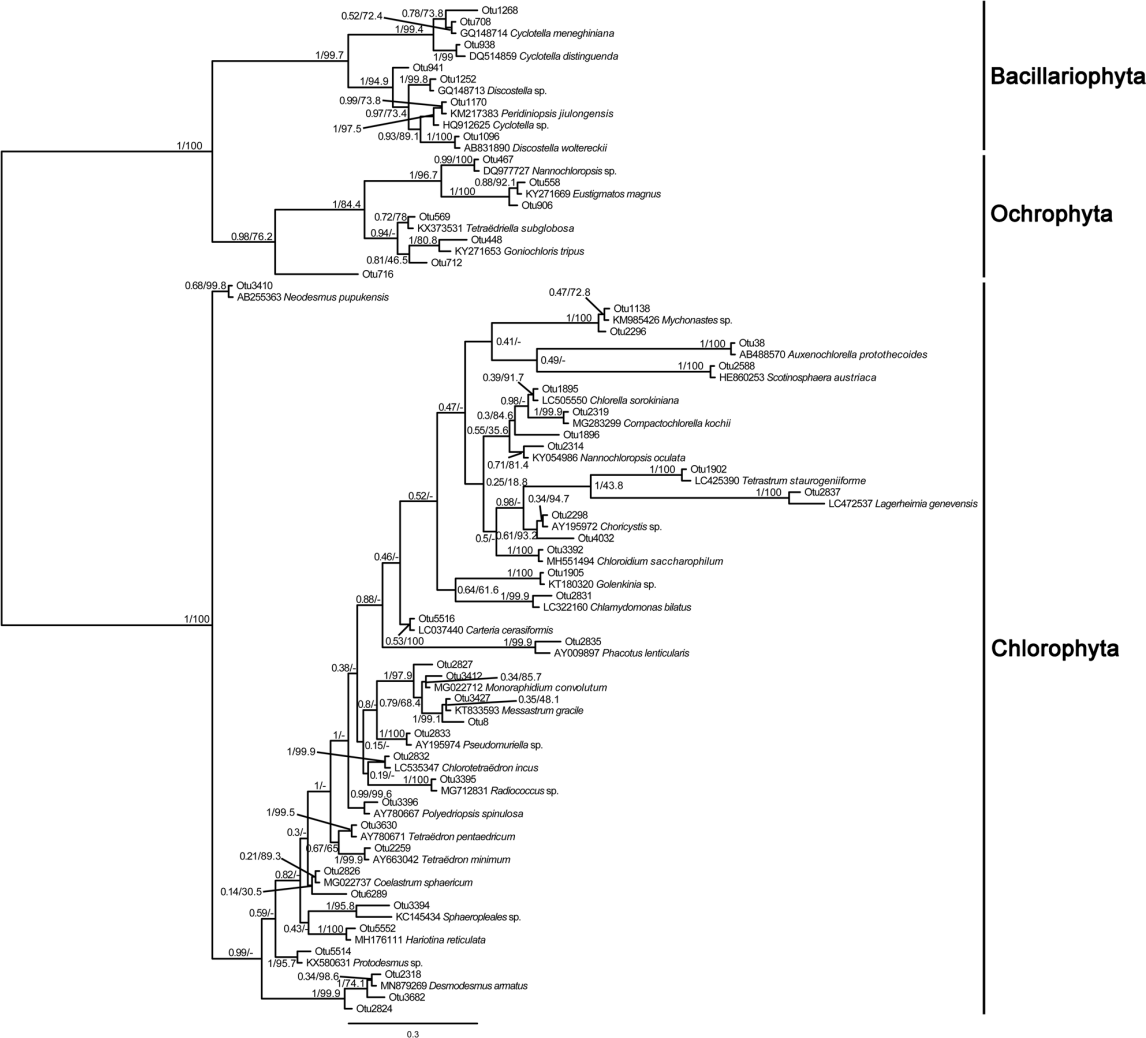
Phylogenetic analysis of the 50 most abundant OTUs in spring, summer, and autumn. Numbers on the left side at the branches represent Bayesian test support values and right side at the branches represent maximum-likelihood bootstrap values.

**Figure 5:**
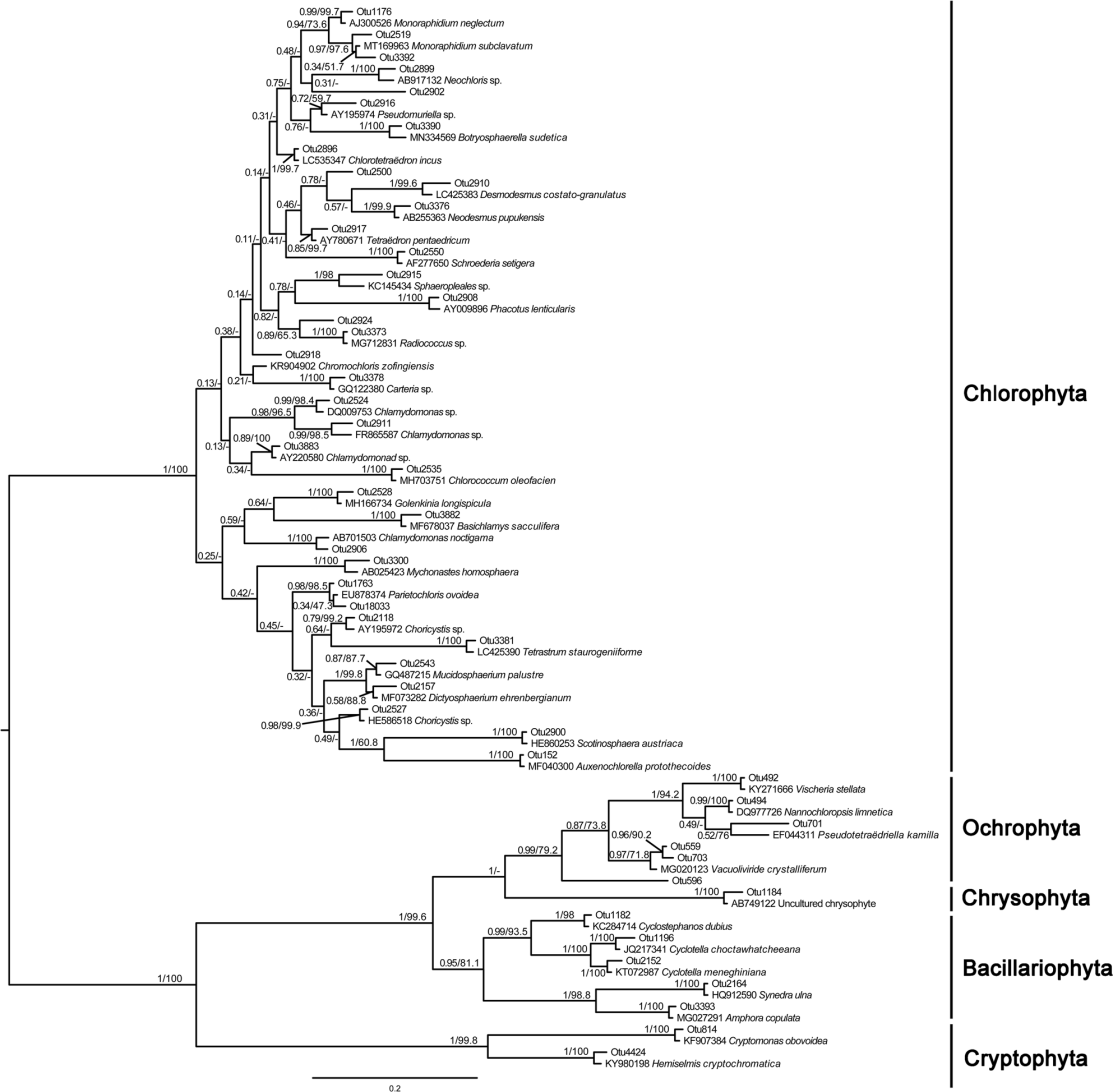
Phylogenetic analysis of the 50 most abundant OTUs in winter. Numbers on the left side at the branches represent Bayesian test support values and right side at the branches represent maximum-likelihood bootstrap values.

In this study, the first 50 representative sequences of samples collected in winter were different from those of samples collected in other seasons. Therefore, two phylogenetic evolution trees were constructed (Figs. 4 & 5). Firstly, we used the 50 most abundant OTUs to blast against the NCBI database, downloaded each of the most relevant sequences with the clearest taxonomic annotations, and then clustered them with our 50 OTUs. As shown in Fig. 4, 36 OTUs in Chlorophyta formed a monophyletic clade with sequences belonging to 27 genera. In Bacillariophyta, 7 OTUs (OTU1268, OTU708, OTU938, OTU941, OTU1252, OTU1170, and OTU1096) formed a monophyletic clade with sequences belonging to 2 genera, *Cyclotella* and *Discostella*, indicating that the species in these two genera were highly abundant. At the same time, we found that OTU1170 had the closest relationship with the *Peridiniopsis jiulongensis* H.Gu (now is accepted taxonomically as *Unruhdinium jiulongense* (H.Gu) Gottschling) diatom endosymbiont, which suggests that OTU1170 may be a *Peridiniopsis* diatom endosymbiont sequence. Besides, OTU1170 was clustered with OTU1252 and OTU1096, which belonged to *Discostella*. Previous studies have shown that the endosymbionts of *Peridiniopsis* may originate from the *Discostella*-like species^25^, indicating the two are closely related. For the phylogenetic analysis of Ochrophyta, 7 OTUs were closely related to the genera such as *Nannochloropsis*, *Eustigmatos*, *Tetraëdriella*, and *Goniochloris*, which have not or hardly been reported to be present in the Fenhe River, reflecting that there are substantially more species of Ochrophyta in this river.

As illustrated in Fig. 5, the abundance of some genera such as *Neochloris*, *Botryosphaerella*, *Schroederia*, and *Chromochloris* was higher in winter than in other seasons. For Ochrophyta, 6 OTUs formed the monophyletic clade with sequences belonging to 4 genera, including *Vischeria*, *Nannochloropsis*, *Pseudotetraëdriella*, and *Vacuoliviride*. Two OTUs from Cryptophyta were clustered with *Cryptomonas obovoidea* Pascher and *Hemiselmis cryptochromatica* C.E.Lane & J.M.Archibald, respectively, with a support rate of 100%.

**Figure 6:**
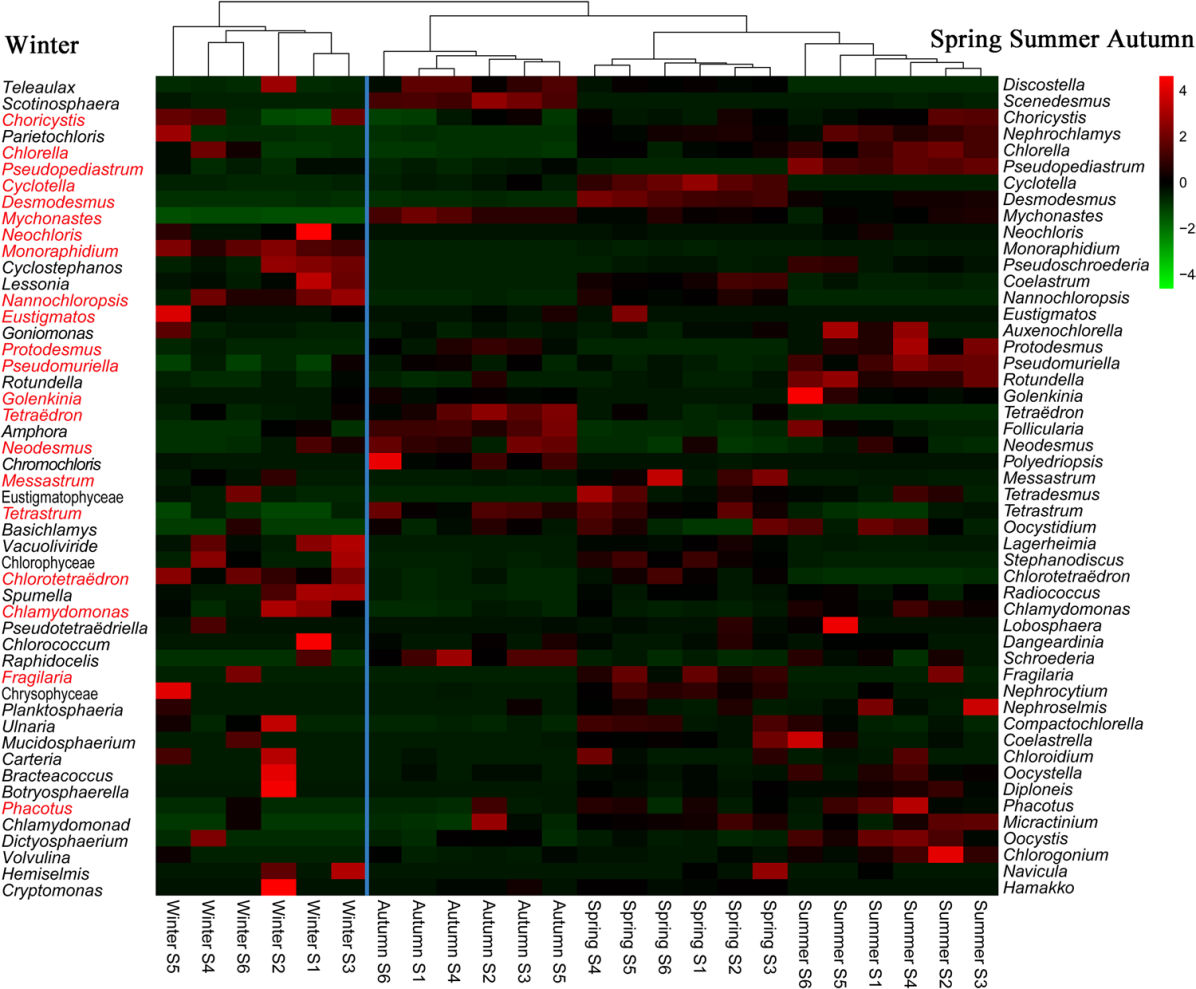
Heatmap of the 50 most abundant genera in all sampling sites based on the relative abundance. One column represents one sample, from left-most column to right-most column is the sites S1–S6 in winter, sites S1–S6 in autumn, sites S1–S6 in spring, sites S1–S6 in summer. The red font indicates the same algal genera in winter as in other seasons.

To find the most widely distributed genera in the Fenhe River, a heatmap showing the relative abundance of 50 genera was plotted (Fig. 6). The heatmap clearly showed that the genus *Desmodesmus*, *Cyclotella*, and *Fragilaria* had the highest relative abundance and were most widely distributed in spring, which is in agreement with the occupancy-abundance relationship. In summer, the average proportions of *Pseudomuriella*, *Pseudopediastrum*, *Rotundella*, and *Oocystis* were higher than those of other genera at the same sites. *Mychonastes*, *Scenedesmus*, *Neodesmus*, *Follicularia*, and *Schroederia* were widely distributed in autumn. The *Cryptomonas* genus at site S2 reached its peak during winter.

### Relationships between phytoplankton communities and physicochemical parameters

The dynamic pattern of phytoplankton communities along the Fenhe River can be influenced by the fluctuations of physical and chemical variables. In general, specific phytoplankton at the different taxonomic levels (mainly phylum, class, order, family, and genus) were related to certain environmental variables (see Supplementary Table S1 online). For instance, at the phylum level, the variations of most members of the Bacillariophyta and Ochrophyta were significantly associated with both physical and chemical properties (*P* < 0.01 and *P* < 0.05). Although the Chlorophyta had no significant correlation with these parameters, at the class level, Chlorophyceae was positively associated with all studied environmental variables, both the physical and chemical properties. Taken together, the phytoplankton communities in the Fenhe River were influenced by the seasonal changes of physical and chemical factors.

To further determine crucial environmental parameters causing the seasonal changes of phytoplankton, we employed Pearson correlation analysis to analyze the relationship between the relative abundance of representative taxa and water quality. Among the effects of environmental variables upon phylotypes at different classification levels, the influence of temperature, phosphate, and DOC on phytoplankton was extensive. For example, the relative abundance of the phyla Bacillariophyta, the class Chlorophyceae, the order Sphaeropleales, and the families Mychonastaceae, Radiococcaceae, Scenedesmaceae, and Selenastraceae was significantly correlated with temperature, phosphate, and DOC concentration (*P* < 0.05). Among these taxa, the family Selenastraceae was significantly negatively correlated with temperature, phosphate, and DOC concentration (*P* < 0.05). Additionally, temperature and phosphate concentration had negative correlations with the relative abundance of the phylum Ochrophyta (including the class Eustigmatophyceae and the order Eustigmatales). Overall, phytoplankton phylotypes associated with the temperature, phosphate, and DOC covered a wider range of taxonomic resolutions than nitrate and nitrite, suggesting that carbon and phosphorus are more likely to be the factors affecting the formation of phytoplankton communities in the Fenhe River than nitrogen.

### Phytoplankton functional groups and driving factors

**Figure 7:**
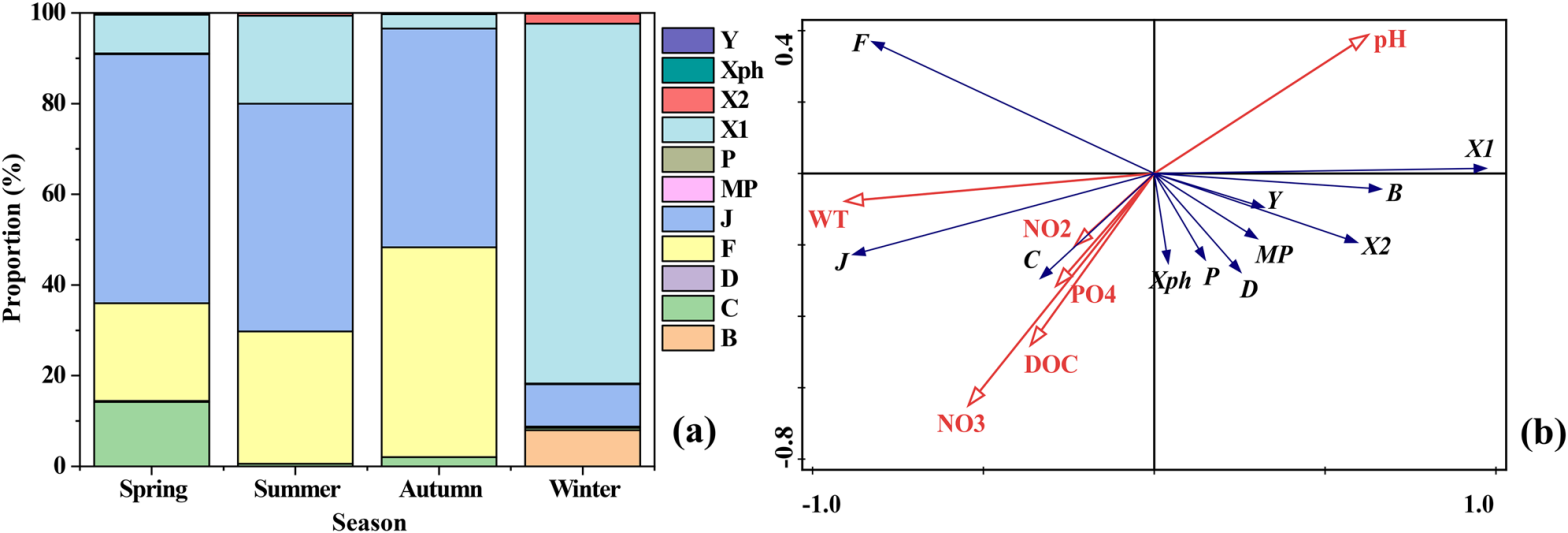
The proportion of the phytoplankton functional groups divided according to the reference ^19,36^ during the four seasons in Fenhe River (**a**); RDA ordination diagram of the phytoplankton functional groups and environmental variables (**b**).

The proportion of phytoplankton functional groups divided according to the references ^19,36^ is shown in Fig. 7. Group J was dominant in spring, summer, and autumn, accounting for 54.97%, 50.25%, and 48.29%, respectively (Fig. 7a). Besides, group F also presented high proportions, its average proportion accounted for 21.54% in spring, 29.18% in summer, and 46.24% in autumn. Group X1 occurred during all seasons, however, the proportion was particularly high in winter. Groups B and X2 were also dominant in winter compared to other seasons.

The RDA ordination diagram of environmental variables and phytoplankton functional groups is shown in Fig. 7b. The first two axes explained 82.89% and 3.87% of the cumulative variance of the relationship of species-environmental variables, and the eigenvalues were 0.8289 and 0.0387, respectively (Table 2). The species-environment correlation values of axis 1 and axis 2 were 0.9807 and 0.6262, respectively. Monte Carlo permutation tests showed that environmental factors such as water temperature (*F* = 46.3, *P* = 0.002), nitrite (*F* = 13.5, *P* = 0.002), and phosphate (*F* = 3.8, *P* = 0.014) were the main factors regulating the phytoplankton functional groups. In Fig. 7b, axis 1 was positively correlated to pH while negatively correlated to water temperature. Axis 2 was negatively correlated with nitrite, phosphate, and DOC. Moreover, groups X1 were greatly affected by pH, and group J showed a strong positive correlation with temperature. Groups C and Xph showed different degrees of positive correlation with phosphate.

**Table 2:** Summary statistics for the first four axes of RDA.

Statistic	Axis 1	Axis 2	Axis 3	Axis 4
Eigenvalues	0.8289	0.0387	0.0098	0.0012
Cumulative % variance of species data	82.89	86.76	87.74	87.86
Species–environmental correlation	0.9807	0.6262	0.6230	0.2993
Cumulative % variance of species–environmental relationship	94.34	98.75	99.86	100

Cluster analysis based on environmental variables (Fig. 1) showed that the upstream and midstream samples were clustered into a branch, however, they were separated from the downstream samples, indicating that the upstream and midstream environments were similar, and showed certain environmental differences from the downstream. The variance partitioning analysis also showed the same spatial difference (Table 3). The pH could explain 41.8% (*P* < 0.01) of the changes in phytoplankton functional groups of upstream and midstream, followed by phosphate (15.6%) and nitrite (10.1%). Water temperature had the largest explanation rate (39.4%) (*P* < 0.05) to phytoplankton functional groups at the downstream of river, followed by phosphate (36.5%), and that the explanation rate of pH was relatively low (12.2%).

**Table 3:** Spatial variance partitioning analysis between phytoplankton functional groups and environmental variables. Bold indicates a difference that is statistically significant (*P* < 0.05).

Upstream and midstream data			Downstream data		
Variables	Explained %	*P*	Variables	Explained %	*P*
pH	41.8	**0.008**	Water temperature	39.4	**0.047**
Phosphate	15.6	**0.036**	Phosphate	36.5	**0.012**
Nitrite	10.1	**0.024**	pH	12.2	**0.040**
Water temperature	6.4	0.216	Nitrate	3.8	0.302
DOC	6.3	0.070	Nitrite	3.3	0.374
Nitrate	0.6	0.786	DOC	0.4	0.836
Unexplained	19.2		Unexplained	4.4	

## Discussion

High-throughput sequencing not only can rapidly and accurately reveal the information of a number of unknown taxa, but also can provide a better understanding of ecological factors that influence their distribution patterns^26^. The 18S rDNA was selected as the target gene because it contains the variable regions that allow detailed classification and highly conserved regions that are required for primer annealing. In addition, it is easy to amplify because of its high copy numbers^27,28^. Thus far, most research has focused on investigating marine environments, but freshwater ecosystems, which also have great biodiversity, are rarely studied. As a result, most sequences in the database are derived from marine microorganisms, causing many freshwater organisms to lack the corresponding OTUs in the SILVA 18S database, thus cannot be classified or assigned annotations. This emphasizes the importance of further research on freshwater environments. In our study, the eukaryotic phytoplankton composition and diversity in the Fenhe River were evaluated based on 18S rDNA, and the effects of seasonal physiochemical factors on the formation of the communities at different taxonomic levels were explored. The results showed the Fenhe River was environmentally heterogeneous, and mainly autotrophic organisms. We also discovered its high diversity value and temporal distribution pattern of phytoplankton. Although the spatial filters had no effect on the diversity, which is in contrary to our initial expectation, the samples from downstream sites were separated from those from the upstream and midstream sites during all the four seasons.

The Fenhe River runs across the urban, agricultural, and industrial areas; thus, the sources of pollutants in surface water at different sampling sites may be different^6,29^. There is less human disturbance and more surrounding aquatic plants (such as reeds) in the upstream area, which have a purifying effect on water quality. Phytoplankton in this area is evenly distributed and has the highest diversity. The downstream area is highly urbanized, densely populated, and is closely related to the good economic development nearby^29^. Domestic sewage is often a potential pollution source in this area, as a result, it had relatively low diversity. In the area in which we studied, the annual precipitation is 538.6 mm, which is concentrated in summer and autumn, but there is less rainfall in spring and winter^30^. In summer, due to the increase of temperature and rainfall, there are more tourists, irrigation and drainage from the surrounding farmlands, causing the water ecological environment to become greatly disturbed, and green algae are prone to outbreaks. After entering autumn, which is a dry season, the water flow velocity decreases; therefore, it is easy to cause individual phytoplankton species to become the dominant species, causing the diversity of phytoplankton and its community structure stability to decline. By contrast, the temperature drops sharply in winter, which is no longer suitable for the growth of most phytoplankton. Less rainfall and lower surface non-point source pollution in spring, and the temperature are suitable for the growth of phytoplankton, which resulting in the increase of its diversity.

Phytoplankton taxa that were significantly different (enriched) were identified by LEfSe analysis, and the frequency and relative abundance of the representative genera were presented by heatmap. Causing the change of phytoplankton communities might be closely associated with local environments. The phytoplankton functional groups have recently been considered as the effective indicator for the understanding of seasonal fluctuations of phytoplankton communities, as they can weaken the individual role of species and explain the response of the phytoplankton communities to environmental changes^31,32^. It is more important and comprehensive than the response of the corresponding individual and population, thus can better reflect the characteristics of the habitat^33^. Factors influencing phytoplankton functional groups usually include water temperature, nutrient availability, and light^34,35^. The changes in water temperature not only can affect the metabolic rate of phytoplankton, but also are related to the degree of dissolution of various nutrients required for growth and reproduction of phytoplankton in water body.

The dominant species in the Fenhe River have both crossover and succession with the seasonal changes. The seasonal succession of phytoplankton functional groups was as follows: spring (J + F + C + X1) → summer (J + F + X1 + X2) → autumn (J + F + X1 + C) → winter (X1 + J + B + X2). Among the main dominant genus (see Supplementary Table S2 online), *Fragilaria* belonging to P group has the highest frequency throughout the year, the P group tends to be strong light and rich nutrients, thus is generally enriched in well-ventilated, nutrient-rich lakes, as well as in the epilimnion of stratified shallow lakes^36^. Group C contained central diatoms, for example, *Cyclotella* sp. (mainly *Cyclotella meneghiniana* Kützing), which was a representative genus with relatively high abundance in spring, which can adjust to small and constantly mixed eutrophication to mesotrophic habitats^19,36^. The growth of most diatoms depends on the availability of nutrients, including carbon, nitrate, silicon, and phosphate, while the increase of TN/TP ratio is related to a higher biomass of group C^37^. The significant increase of carbon and nitrogen concentrations in spring may explain the changes in phytoplankton communities observed here. An increase of nitrogen is usually related to a sudden input of wastewater. As a complex mixture of compounds, DOC is a critical component of carbon transfer from terrestrial to freshwater ecosystems, as well as from sources to marine areas^38^. It has been determined that the subtle changes of DOC concentrations can cause shifts in the structure of aquatic microorganisms^39^. One of the main terrestrial inputs of DOC comes from vegetation, such as the decomposition of organic litter and the direct input of submerged plants^40^, which can release a large amount of DOC into rivers.

It is well known that the trophic status and nutrient concentrations of lakes have different effects on the types of microorganisms^41^. Nitrogen and/or phosphorus are usually the factors that limit the growth of phytoplankton^42^. Chlamydomonadales mainly participate in the remineralization of nitrogen and phosphate. In our study, the family Chlamydomonadaceae and the genus *Chlamydomonas* belonging to X2 group showed significant negative correlation with temperature and phosphate, and had high abundance in winter, which was able to tolerate stratification, but was sensitive to mixing and filter-feeding grazers. Group X1 included small and slender single-celled green algae (*Monoraphidium* sp. and *Chlorella* sp.), they could adjust to low light and were sensitive to nutrient deficiency^19^. Correlation analysis and RDA analysis showed significant negative relationships between group X1 and nutrients (nitrogen and phosphorus) concentrations. The widespread distribution pattern of *Cryptomonas* belonging to Y group can be related to its growth in a variety of environments, as it has been found existed in rivers, reservoirs, and lakes. At the same time, the distribution of this genus does not have any seasonal specificity, because it could occur in the cool winter or bloom in summer^43^. Besides, it is mainly distributed in moderately enriched systems with a high surface, volume ratio, relatively fast growth, and rapid phosphorus uptake rates^44^. According to the literature^45^, the Y group is tolerant to high light attenuation coefficient values, suggesting that it can adjust to the environments with insufficient light. In our study, this group was found in low light conditions during winter. Other studies have also shown that *Cryptomonas* has the ability to enhance nutrient absorption through mixotrophy, the mechanism that enables it to grow and reproduce under a light-limited environment^46^. In addition, it has strengthened competitiveness due to its flagella, which allows it to migrate vertically between water layer with optimal light conditions and nutrient concentrations.

Organisms often have an indicating effect on temperature changes, especially for those residing in aquatic environments, where the range of temperature changes is smaller than that on land, which causing aquatic organisms lack a temperature compensation mechanism, therefore, there are more thermophilic organisms. Correlation analysis demonstrated that diatoms, especially for Bacillariophyta and *Discostella*, were sensitive to temperature changes. The class Chlorophyceae, the order Sphaeropleales, the family Mychonastaceae, and the genus *Mychonastes* and *Tetrastrum* have specific requirements for environmental factors (high temperature, high contents of carbon, nitrogen, and phosphorus), all of which belonged to a widespread species group. The Sphaeropleales (“Chlorococcales”) is a diverse taxon that responds positively to increasing thermal stability by adopting the inclusive range of morphological adaptions to contrast large subsidence losses, for example, small size and mucus formation^47,48^. However, other organisms residing in a cold environment, *Chlorotetraëdron*, *Neochloris*, and *Nannochloropsis* has lower requirements for nutrients and carbon, and are mainly distributed in areas with low temperature.

## Conclusion

This study combined the diversity with functional groups to identify factors driving the seasonal composition of phytoplankton communities in the Fenhe River. The 18S rDNA V4 region revealed that the diversity and composition of phytoplankton varied with seasons, and the diversity and number of species in spring were significantly higher than that in other seasons. LEfSe analysis identified the potential seasonal and spatial biomarkers and detected four to seven phytoplankton taxa each season. Besides, analysis of the systemic evolution and distribution characteristics of the first 50 representative sequences showed that the dominant genus in spring was *Desmodesmus*, whereas that in summer, autumn, and winter was *Pseudopediastrum*, *Mychonastes*, and *Monoraphidium*, respectively. The phytoplankton functional groups have both crossover and succession with the seasonal changes. RDA analysis demonstrated that water temperature, nitrite, and phosphate were considered as the critical factors affecting this fluctuation. The variance partitioning result showed that pH had the largest explanation rate to phytoplankton functional groups of upstream and midstream, water temperature had the largest explanation rate to phytoplankton functional groups at the downstream of river. Pearson correlation analysis indicated that carbon and phosphorus were more likely the determinants than nitrogen in the process of shaping the phytoplankton communities in the Fenhe River. Taken together, these results greatly enhance our knowledge on the spatiotemporal dynamics and diversity of this ecologically significant but little-known urban river.

## Methods

**Figure 8:**
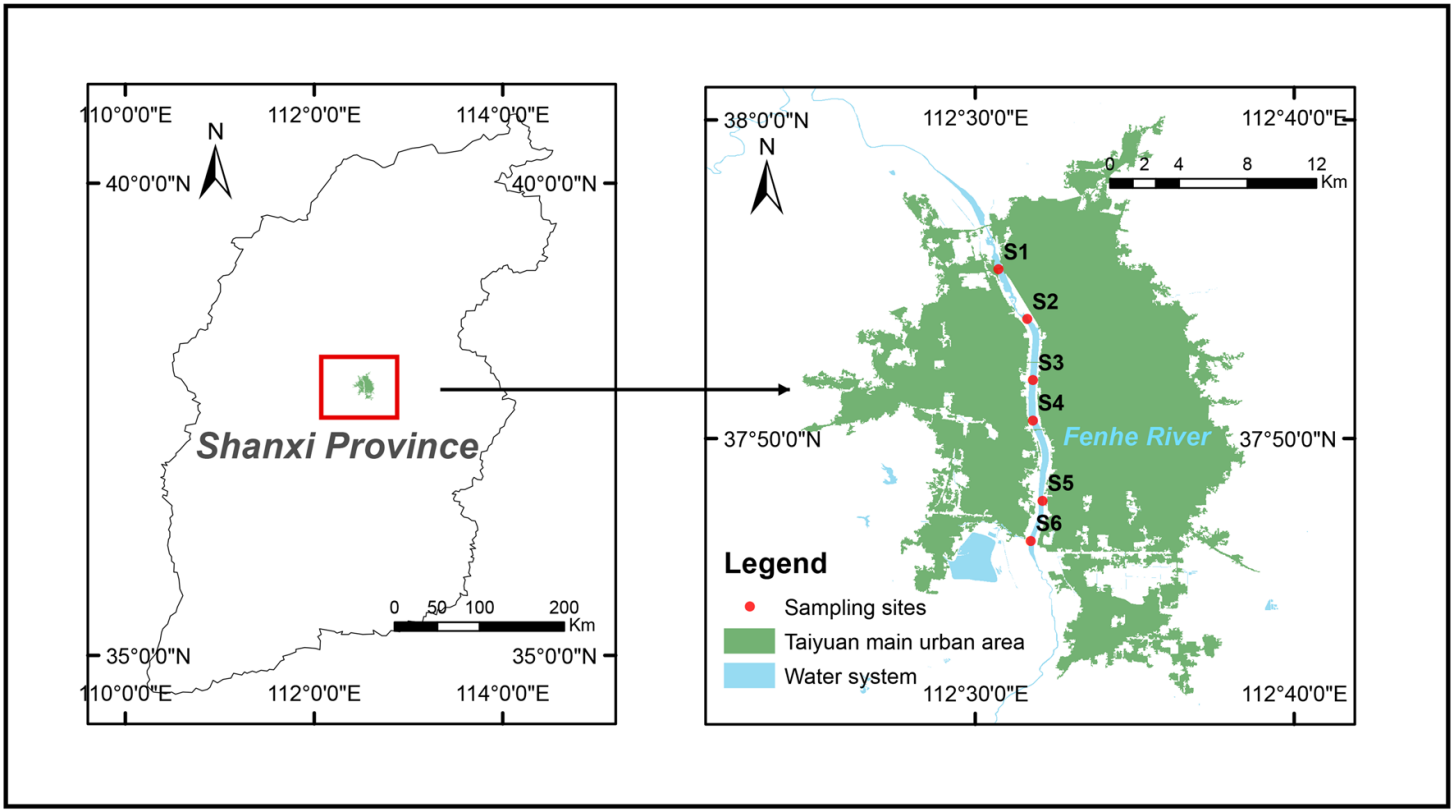
The location of the sampling sites in Fenhe River. The map is produced using GIS software ArcMap (version 10.2) (https://developers.arcgis.com/).

The Fenhe River is the largest river in Shanxi Province, with a catchment area of 39 471 km^2^ and a total length of about 710 km. The study area is located in Taiyuan (latitude: 37°27′–38°25′, longitude: 111°30′–113°09′), Shanxi Province, northern China (Fig. 8). The Taiyuan section of the Fenhe River is a length of 188 km, flows through Taiyuan from north to south, and receives domestic sewage and industrial wastewater discharged along the bank. At the same time, its upstream is also an important supply area for Taiyuan's drinking water source. The urban section of river starts from Chaicun Bridge in the north and reaches at Xiangyun Bridge in the south, with a total length of 20 km; and includes many factories and enterprises, such as coal mines, thermal power stations, coal preparation plants, etc. Therefore, a large amount of untreated industrial sewage is discharged into the river, which is often a potential source of pollution for water bodies in the area. According to statistics, the industrial production and residential water consumption in the Fenhe River is about 2.32 × 10^9^ m^3^/a, which accounts for 46% of the total water consumption in Shanxi Province. And the city is densely populated and highly urbanized, with a population of about 4.5 million. In addition, the region has a semi-humid continental monsoon climate with an average annual precipitation of 538.6 mm. According to the Shanxi Provincial Hydrology and Water Resources Survey Bureau, the water flows of the Fenhe River range from 2.12 to 9.44 m^3^/s, and the water velocities range between 0.35 to 0.58 m/s. The six sampling sites selected in this study are mainly located in the upstream and downstream of the Taiyuan section of the river, as well as the industrial or urban wastewater discharge outlets. The map of Fenhe River is produced using GIS software ArcMap (version 10.2) (https://developers.arcgis.com/).

To investigate the influence of seasonal factors, water samples were taken in March (spring), July (summer), September (autumn), and December (winter) of 2019. The distribution of sampling sites is shown in Fig. 7. The phytoplankton community diversity of the water samples collected from each site was analyzed based on the V4 region of 18S rDNA, and the environmental parameters of the site were measured within 24 h. Water temperature was measured in situ using a multi-parameter water quality monitor, and the concentrations of nitrate (NO_3_^-^), nitrite (NO_2_^-^), and phosphate (PO_4_^3-^) were determined according to the APHA standard methods^49^. The dissolved organic carbon (DOC) concentration was determined according to the method described by Bolan et al.^50^. All experiments were performed in triplicate.

For the analysis of marker gene, 250 mL of water samples were concentrated using an isopore membrane filter (0.22 μm) and then stored at − 80 °C until subsequent DNA extraction. Genomic DNA was extracted using an E.Z.A.N. Mag-Bind DNA Kit according to the manufacturer’s instructions. The V4 region of 18S rDNA was amplified using primers V4F (5'-GGCAAGTCTGGTGCCAG-3') and V4R (5'-ACGGTATCTRATCRTCTTCG-3'), and the PCR amplification was performed according to Sun et al.^51^. The PCR amplification cycle was: the first round of amplification was 94 °C for 3 min, followed by 5 cycles at 94 °C for 30 s, 45 °C for 20 s, 65 °C for 30 s, then followed by 20 cycles at 94 °C for 20 s, 55 °C for 20 s, 72 °C for 30 s, and a final extension at 72 °C for 5 min; the second round of amplification was 95 °C for 3 min, followed by 5 cycles at 94 °C for 20 s, 55 °C for 20 s, 72 °C for 30 s, and a final extension at 72 °C for 5 min. Two round PCR reactions were performed in a 30 μL reaction, containing 15 μL 2 × Taq master Mix, 10 μM of forward and reverse primers, and 20 ng of genomic DNA or PCR products of first-round amplification, respectively.

The quality of raw data was controlled using Qiime 1.7.0. The barcode and adapter sequences were removed by Cutadapt^52^. The chimera and non-specific amplified sequences were removed by Usearch to obtain the effective tags. All the effective tags from each sample were clustered using Usearch software, and the sequences with 97% identity were clustered as Operational Taxonomic Units (OTUs). Selected the top 50 most abundant OTUs as the representative sequences and blast against the nt database in NCBI. Then download the most related sequence with clear taxonomy annotations, and cluster with our 50 OTUs. PHYML software and MrBayes version 3.1.2 were executed to construct the Maximum Likelihood and Bayesian trees, respectively.

The linear discriminant analysis (LDA) effect size (LEfSe) analysis was carried out using a normalized relative abundance matrix (http://huttenhower.sph.harvard.edu/lefse/) to detect the potential biomarkers. The LEfSe method based on the Kruskal–Wallis test was employed to identify features based on seasons and sites that were significant differences, and the LDA was used to assess the effect size of each feature. The significant *P*-value less than 0.05 and the LDA threshold score of 2.0 were used as criteria for identifying biomarkers. The diversity index and richness were analyzed using one-way analysis of variance (ANOVA). Post hoc comparisons were made using Tukey's method, a value of *P* < 0.05 was considered to be significant. Hierarchical cluster analysis of environmental variables based on the Euclidean distance and Ward linkage was conducted by R version 3.5.1 software. Heatmap generated using R version 3.5.1 software was used to detect the distribution of phytoplankton. SPSS 26.0 software was used to perform ANOVA variance analysis to identify the differences between seasonal variables and determine the Pearson correlations between the dominant groups at the different taxonomic levels and the physicochemical characteristics of water.

In this study, the taxa were classified into phytoplankton functional groups according to Reynolds et al.^19^ and Padisák et al.^36^. The relationship between the phytoplankton functional groups and environmental factors was determined by multivariate analysis (Canoco 5.0). Before the analysis, all the phytoplankton functional groups and environmental factors (except for pH) were converted into log_10_ (x + 1). The detrended correspondence analysis (DCA) was employed to decide whether a linear or unimodal ordination method should be applied. The results demonstrated that the maximum value of the four-axis length gradient was 2.1, and linear model RDA was performed to identify the effects of environmental variables on phytoplankton functional groups. Prior to conducting the RDA analysis, Monte Carlo tests were used to screen out those environmental variables that had significant impacts on the phytoplankton functional groups. On the basis of the RDA analysis results, variance partitioning analysis (Canoco 5.0) was carried out to evaluate the spatial effects of the environmental parameters on the changes in phytoplankton functional groups.

## Supplementary Information

Supplementary Information.

## Data Availability

The datasets generated during and/or analysed during the current study are available in the NCBI repository (accession: PRJNA648254).
