# Supplementary material for: Seasonal and spatial patterns of eukaryotic phytoplankton communities in an urban river based on marker gene
Source: Sci Rep. 2021 Nov 30;11:23147. doi: 10.1038/s41598-021-02183-5 (PMC8633359; doi:10.1038/s41598-021-02183-5)
Supplement: Supplementary file 1 — Supplementary Information. [file 41598_2021_2183_MOESM1_ESM.doc]

Seasonal and spatial patterns of eukaryotic phytoplankton communities in an urban river based on marker gene

Jing Yang ^1^, Junping Lv ^1^, Qi Liu ^1^, Fangru Nan ^1^, Bo Li ^2^, Shulian Xie ^1^ & Jia Feng ^1^*

^1^School of Life Science, Shanxi University, Taiyuan 030006, China

^2^School of Geographical Science, Taiyuan Normal University, Jinzhong 030619, China

*Corresponding author: (e-mail: [fengj@sxu.edu.cn](mailto:fengj@sxu.edu.cn))

**Supplementary Table S1** Pearson correlation between relative abundance of dominate phytoplankton groups at different taxonomic levels and physiochemical parameters

|  | Taxonomic | Water temperature | | Nitrate | | Nitrite | | Phosphate | | DOC | |
| --- | --- | --- | --- | --- | --- | --- | --- | --- | --- | --- | --- |
|  |  | r | *P* | r | *P* | r | *P* | r | *P* | r | *P* |
| Phylum | Bacillariophyta | .518^**^ | **0.010** | .655^**^ | **0.001** | 0.195 | 0.360 | .461^*^ | **0.023** | .554^**^ | **0.005** |
|  | Ochrophyta | -.477^*^ | **0.018** | 0.035 | 0.870 | 0.262 | 0.216 | 0.183 | 0.392 | -.554^**^ | **0.005** |
| Class | Bacillariophyceae | -0.041 | 0.849 | -0.255 | 0.229 | -.458^*^ | **0.024** | -0.398 | 0.054 | 0.027 | 0.901 |
|  | Mediophyceae | 0.328 | 0.117 | .545^**^ | **0.006** | 0.174 | 0.415 | .493^*^ | **0.014** | 0.367 | 0.078 |
|  | Chlorophyceae | .929^**^ | **0.000** | .532^**^ | **0.007** | .473^*^ | **0.020** | .428^*^ | **0.037** | .907^**^ | **0.000** |
|  | Trebouxiophyceae | 0.106 | 0.623 | -0.117 | 0.585 | -.432^*^ | **0.035** | -.477^*^ | **0.018** | 0.212 | 0.320 |
|  | Eustigmatophyceae | -.775^**^ | **0.000** | -0.244 | 0.251 | -0.277 | 0.189 | -0.238 | 0.263 | -.780^**^ | **0.000** |
| Order | Stephanodiscales | 0.328 | 0.118 | .545^**^ | **0.006** | 0.175 | 0.414 | .494^*^ | **0.014** | 0.366 | 0.078 |
|  | Sphaeropleales | .895^**^ | **0.000** | .718^**^ | **0.000** | .589^**^ | **0.002** | .597^**^ | **0.002** | .886^**^ | **0.000** |
|  | Chlorellales | 0.358 | 0.086 | 0.028 | 0.897 | -.440^*^ | **0.031** | -0.392 | 0.058 | .422^*^ | **0.040** |
|  | Trebouxiophyceae ordo incertae sedis | -0.148 | 0.491 | -0.081 | 0.708 | -0.197 | 0.356 | -.469^*^ | **0.021** | -0.118 | 0.582 |
|  | Eustigmatales | -.805^**^ | **0.000** | -0.260 | 0.220 | -.432^*^ | **0.035** | -0.331 | 0.115 | -.766^**^ | **0.000** |
|  | Goniochloridales | 0.054 | 0.801 | 0.050 | 0.815 | .691^**^ | **0.000** | .407^*^ | **0.048** | -0.147 | 0.493 |
| Family | Stephanodiscaceae | 0.328 | 0.118 | .545^**^ | **0.006** | 0.175 | 0.414 | .494^*^ | **0.014** | 0.366 | 0.078 |
|  | Mychonastaceae | .862^**^ | **0.000** | .525^**^ | **0.008** | .642^**^ | **0.001** | .742^**^ | **0.000** | .776^**^ | **0.000** |
|  | Chlamydomonadaceae | -0.177 | 0.408 | -0.162 | 0.451 | -0.290 | 0.169 | -.564^**^ | **0.004** | -0.131 | 0.540 |
|  | Neochloridaceae | -.669^**^ | **0.000** | -0.340 | 0.105 | -0.283 | 0.181 | -0.160 | 0.455 | -.654^**^ | **0.001** |
|  | Sphaeropleales incertae sedis | 0.310 | 0.140 | 0.132 | 0.539 | .706^**^ | **0.000** | .506^*^ | **0.012** | 0.189 | 0.378 |
|  | Pseudomuriellaceae | .411^*^ | **0.046** | 0.031 | 0.886 | -0.017 | 0.936 | -0.296 | 0.160 | 0.366 | 0.079 |
|  | Radiococcaceae | .685^**^ | **0.000** | 0.035 | 0.872 | .491^*^ | **0.015** | .422^*^ | **0.040** | .565^**^ | **0.004** |
|  | Rotundellaceae | 0.328 | 0.118 | -0.147 | 0.492 | -0.160 | 0.454 | -0.327 | 0.119 | 0.330 | 0.115 |
|  | Scenedesmaceae | .769^**^ | **0.000** | .836^**^ | **0.000** | 0.273 | 0.197 | .432^*^ | **0.035** | .826^**^ | **0.000** |
|  | Selenastraceae | -.952^**^ | **0.000** | -.562^**^ | **0.004** | -0.359 | 0.085 | -.470^*^ | **0.021** | -.890^**^ | **0.000** |
|  | Chlorellaceae | 0.139 | 0.516 | -0.084 | 0.697 | -.408^*^ | **0.048** | -.491^*^ | **0.015** | 0.174 | 0.415 |
|  | Oocystaceae | .444^*^ | **0.030** | 0.092 | 0.667 | -0.401 | 0.052 | -0.279 | 0.186 | .518^**^ | **0.010** |
|  | Coccomyxaceae | -0.184 | 0.390 | -0.099 | 0.646 | -0.166 | 0.438 | -.437^*^ | **0.033** | -0.163 | 0.448 |
|  | Monodopsidaceae | -.737^**^ | **0.000** | -0.232 | 0.274 | -.452^*^ | **0.027** | -0.344 | 0.100 | -.737^**^ | **0.000** |
|  | Goniochloridaceae | 0.054 | 0.801 | 0.050 | 0.815 | .691^**^ | **0.000** | .407^*^ | **0.048** | -0.147 | 0.493 |
| Genus | *Cyclotella* | 0.289 | 0.171 | .549^**^ | **0.005** | -0.237 | 0.265 | 0.059 | 0.784 | .442^*^ | **0.030** |
|  | *Discostella* | .493^*^ | **0.014** | 0.369 | 0.076 | .618^**^ | **0.001** | .816^**^ | **0.000** | 0.392 | 0.058 |
|  | *Chlamydomonas* | -.439^*^ | **0.032** | -0.221 | 0.300 | -0.333 | 0.112 | -.502^*^ | **0.012** | -0.383 | 0.065 |
|  | *Tetraëdron* | 0.283 | 0.180 | 0.365 | 0.080 | .778^**^ | **0.000** | .616^**^ | **0.001** | 0.146 | 0.497 |
|  | *Mychonastes* | .862^**^ | **0.000** | .525^**^ | **0.008** | .642^**^ | **0.001** | .742^**^ | **0.000** | .776^**^ | **0.000** |
|  | *Chlorotetraëdron* | -.739^**^ | **0.000** | -0.274 | 0.196 | -0.299 | 0.156 | -0.323 | 0.124 | -.629^**^ | **0.001** |
|  | *Neochloris* | -.439^*^ | **0.032** | -0.265 | 0.210 | -0.191 | 0.370 | -0.028 | 0.898 | -.472^*^ | **0.020** |
|  | *Polyedriopsis* | 0.310 | 0.140 | 0.132 | 0.539 | .706^**^ | **0.000** | .506^*^ | **0.012** | 0.189 | 0.378 |
|  | *Pseudomuriella* | .411^*^ | **0.046** | 0.031 | 0.886 | -0.017 | 0.936 | -0.296 | 0.160 | 0.366 | 0.079 |
|  | *Follicularia* | .539^**^ | **0.007** | -0.011 | 0.958 | .651^**^ | **0.001** | .543^**^ | **0.006** | 0.376 | 0.070 |
|  | *Radiococcus* | .483^*^ | **0.017** | 0.124 | 0.563 | -0.366 | 0.079 | -0.266 | 0.209 | .582^**^ | **0.003** |
|  | *Coelastrum* | 0.245 | 0.248 | .509^*^ | **0.011** | -0.333 | 0.111 | 0.001 | 0.998 | 0.403 | 0.051 |
|  | *Desmodesmus* | .421^*^ | **0.040** | .533^**^ | **0.007** | -0.400 | 0.053 | -0.132 | 0.539 | .586^**^ | **0.003** |
|  | *Neodesmus* | 0.190 | 0.374 | -0.109 | 0.611 | .602^**^ | **0.002** | .549^**^ | **0.006** | -0.005 | 0.980 |
|  | *Protodesmus* | .419^*^ | **0.041** | 0.116 | 0.591 | 0.325 | 0.121 | -0.071 | 0.743 | 0.344 | 0.099 |
|  | *Scenedesmus* | .431^*^ | **0.035** | 0.363 | 0.081 | .917^**^ | **0.000** | .720^**^ | **0.000** | 0.285 | 0.177 |
|  | *Tetradesmus* | 0.315 | 0.134 | .526^**^ | **0.008** | -0.326 | 0.121 | -0.139 | 0.516 | .441^*^ | **0.031** |
|  | *Tetrastrum* | .589^**^ | **0.002** | .704^**^ | **0.000** | .526^**^ | **0.008** | .555^**^ | **0.005** | .565^**^ | **0.004** |
|  | *Monoraphidium* | -.952^**^ | **0.000** | -.577^**^ | **0.003** | -0.346 | 0.098 | -.463^*^ | **0.023** | -.896^**^ | **0.000** |
|  | *Chlorella* | 0.029 | 0.894 | -0.127 | 0.554 | -.437^*^ | **0.033** | -.540^**^ | **0.006** | 0.064 | 0.768 |
|  | *Nephrochlamys* | .430^*^ | **0.036** | 0.077 | 0.719 | -0.404 | 0.051 | -0.276 | 0.192 | .502^*^ | **0.012** |
|  | *Oocystella* | 0.398 | 0.054 | 0.074 | 0.730 | -0.179 | 0.403 | -0.361 | 0.083 | .417^*^ | **0.043** |
|  | *Choricystis* | .642^**^ | **0.001** | .452^*^ | **0.027** | 0.078 | 0.719 | -0.063 | 0.771 | .671^**^ | **0.000** |
|  | *Nannochloropsis* | -.737^**^ | **0.000** | -0.232 | 0.274 | -.452^*^ | **0.027** | -0.344 | 0.100 | -.737^**^ | **0.000** |
|  | *Vacuoliviride* | -.460^*^ | **0.024** | -0.298 | 0.158 | 0.180 | 0.401 | 0.120 | 0.577 | -.637^**^ | **0.001** |

** denotes *P*<0.01, * denotes *P*<0.05

**Supplementary Table S2** The representative taxa of phytoplankton functional groups and their appearance season in the Fenhe River

| Genera | Taxonomic group | Functional group | Species | Spring | Summer | Autumn | Winter |
| --- | --- | --- | --- | --- | --- | --- | --- |
| *Cyclostephanos* | Bacillariophyta | B | *Cyclostephanos dubius* (Hustedt) Round |  |  |  | √ |
| *Cyclotella* | Bacillariophyta | C | *Cyclotella meneghiniana* Kützing | √ | √ | √ | √ |
| *Ulnaria* | Bacillariophyta | D | *Ulnaria acus* (Kützing) Aboal | √ |  | √ |  |
| *Ulnaria* | Bacillariophyta | MP | *Ulnaria oxyrhynchus* (Kützing) Aboal |  | √ | √ | √ |
| *Stephanodiscus* | Bacillariophyta | D | *Stephanodiscus hantzschii* Grunow in Cleve & Grunow | √ |  |  |  |
| *Stephanodiscus* | Bacillariophyta | C | *Stephanodiscus minutulus* (Kützing) Cleve & Möller | √ | √ |  |  |
| *Micractinium* | Chlorophyta | F | *Micractinium pusillum* Fresenius | √ | √ | √ |  |
| *Micractinium* | Chlorophyta | F | *Micractinium belenophorum* (Korshikov) T.Proschold, C.Block, W.Luo & L.Kreinitz | √ | √ | √ |  |
| *Micractinium* | Chlorophyta | F | *Micractinium inermum* R.Hoshina & Y.Fujiwara | √ |  |  |  |
| *Nephrocytium* | Chlorophyta | F | *Nephrocytium agardhianum* Nägeli | √ | √ | √ |  |
| *Oocystis* | Chlorophyta | F | *Oocystis* sp. |  |  | √ |  |
| *Oocystis* | Chlorophyta | F | *Oocystis parva*West & G.S.West | √ | √ |  |  |
| *Dictyosphaerium* | Chlorophyta | F | *Dictyosphaerium lacustre* C.Bock, Proschold & Krienitz | √ |  |  |  |
| *Dictyosphaerium* | Chlorophyta | F | *Dictyosphaerium* sp. |  | √ | √ |  |
| *Dictyosphaerium* | Chlorophyta | F | *Dictyosphaerium ehrenbergianum*Nägeli |  |  |  | √ |
| *Planktosphaeria* | Chlorophyta | F | *Planktosphaeria* sp. | √ |  |  |  |
| *Planktosphaeria* | Chlorophyta | F | *Planktosphaeria gelatinosa* G.M.Smith |  |  |  | √ |
| *Oocystidium* | Chlorophyta | F | *Oocystidium polymammilatum* (latest *Echinocoleum polymammilatum* (Hortobágyi) Hindák & Horecká) | √ | √ | √ |  |
| *Mychonastes* | Chlorophyta | F | *Mychonastes ovahimbae* Krienitz, C.Bock, Dadheech & Proschold | √ |  | √ |  |
| *Mychonastes* | Chlorophyta | F | *Mychonastes homosphaera*(Skuja) Kalina & Puncochárová |  |  | √ | √ |
| *Mychonastes* | Chlorophyta | F | *Mychonastes jurisii*(Hindák) Krienitz, C.Bock, Dadheech & Proschold | √ | √ | √ |  |
| *Desmodesmus* | Chlorophyta | J | *Desmodesmus armatus* (Chodat) E.H.Hegewald | √ |  |  |  |
| *Desmodesmus* | Chlorophyta | J | *Desmodesmus abundans*(Kirchner) E.H.Hegewald | √ | √ | √ |  |
| *Desmodesmus* | Chlorophyta | J | *Desmodesmus denticulatus*(Lagerheim) S.S.An, T.Friedl & E.Hegewald | √ | √ | √ |  |
| *Desmodesmus* | Chlorophyta | J | *Desmodesmus communis*(E.Hegewald) E.Hegewald | √ |  | √ |  |
| *Coelastrum* | Chlorophyta | J | *Coelastrum microporum* Nägeli | √ | √ | √ |  |
| *Pseudopediastrum* | Chlorophyta | J | *Pseudopediastrum alternans* (Nygaard) M.Jena & C.Bock | √ | √ | √ | √ |
| *Chlorotetraëdron* | Chlorophyta | J | *Chlorotetraedron incus* (Teiling) Komárek & Kovácik | √ | √ | √ | √ |
| *Tetrastrum* | Chlorophyta | J | *Tetrastrum staurogeniiforme* (Schröder) Lemmermann | √ | √ | √ | √ |
| *Tetraëdron* | Chlorophyta | J | *Tetraëdron minimum* (A.Braun) Hansgirg | √ | √ | √ |  |
| *Tetraëdron* | Chlorophyta | J | *Tetraëdron caudatum*(Corda) Hansgirg | √ | √ | √ |  |
| *Tetraëdron* | Chlorophyta | J | *Tetraëdron pentaedricum*West & G.S.West |  |  |  | √ |
| *Scenedesmus* | Chlorophyta | J | *Scenedesmus littoralis*Hanagata | √ |  |  |  |
| *Lagerheimia* | Chlorophyta | J | *Lagerheimia genevensis* (Chodat) Chodat | √ | √ |  |  |
| *Golenkinia* | Chlorophyta | J | *Golenkinia paucispina*West & G.S.West |  | √ | √ |  |
| *Fragilaria* | Bacillariophyta | P | *Fragilaria nanana* Lange-Bertalot | √ | √ |  | √ |
| *Chlorella* | Chlorophyta | X1 | *Chlorella vulgaris* Beijerinck | √ | √ |  |  |
| *Choricystis* | Chlorophyta | X1 | *Choricystis* sp./ Choricystis parasitica (K.Brandt) Pröschold & Darienko | √ | √ | √ | √ |
| *Monoraphidium* | Chlorophyta | X1 | *Monoraphidium minutum* (Nägeli) Komárková-Legnerová | √ | √ | √ | √ |
| *Pseudoschroederia* | Chlorophyta | X1 | *Pseudoschroederia antillarum* (Komárek) Hegewald & Schnepf | √ | √ | √ |  |
| *Auxenochlorella* | Chlorophyta | X1 | *Auxenochlorella protothecoides* (Krüger) Kalina & Puncochárová | √ | √ | √ |  |
| *Schroederia* | Chlorophyta | X1 | *Schroederia setigera* (Schröder) Lemmermann | √ | √ | √ |  |
| *Chlamydomonas* | Chlorophyta | X2 | *Chlamydomonas bilatus* Ettl | √ | √ | √ |  |
| *Chlorogonium* | Chlorophyta | X2 | *Chlorogonium complexum* Nakada |  | √ | √ |  |
| *Chlorogonium* | Chlorophyta | X2 | *Chlorogonium* sp. | √ |  | √ |  |
| *Cryptomonas* | Cryptophyta | Y | *Cryptomonas obovoidea* Pascher |  |  |  | √ |
| *Chlorococcum* | Chlorophyta | MP | *Chlorococcum oleofaciens* Trainor & Bold | √ |  |  | √ |
| *Navicula* | Bacillariophyta | MP | *Navicula trivialis* Lange-Bertalot | √ |  |  |  |
| *Phacotus* | Chlorophyta | Xph | *Phacotus lenticularis* (Ehrenberg) Diesing |  | √ | √ | √ |
